# State of the Art Membrane Science and Technology in the Iberian Peninsula 2021–2022

**DOI:** 10.3390/membranes13080732

**Published:** 2023-08-15

**Authors:** Clara Casado-Coterillo, Diogo M. F. Santos, Liliana C. Tomé, Svetlozar Velizarov, Isabel Coelhoso, José Ignacio Calvo

**Affiliations:** 1 Department of Chemical and Biomolecular Engineering, University of Cantabria, Av. Los Castros s/n, 39005 Santander, Spain; 2 Center of Physics and Engineering of Advanced Materials, Laboratory for Physics of Materials and Emerging Technologies, Chemical Engineering Department, Instituto Superior Técnico, Universidade de Lisboa, 1049-001 Lisbon, Portugal; 3 LAQV/REQUIMTE, Department of Chemistry, NOVA School of Science and Technology, Universidade Nova de Lisboa, 2829-516 Caparica, Portugal; 4 Department of Applied Physics, Institute for Sustainable Processes, Universidad de Valladolid, 34071 Palencia, Spain

This Special Issue of the journal *Membranes* arises from the need to highlight the developments in the field of membrane research and membrane processes that have been emerging in recent years by researchers and research groups based in the Iberian Peninsula. Despite the absence of membrane companies fabricating and commercializing membranes in the Peninsula until last year, many Portuguese and Spanish researchers have followed the pioneering steps of the membrane science and technology coined in Europe in the 1970s, contributing to the study and application of membranes in existing and new processes relevant to the reality of both Portugal and Spain. This Special Issue is composed of 14 research articles plus 2 reviews, each focusing on the perspective of one country (Spain or Portugal) but adding to the historical perspective of the beginning of membrane technology in each country.

Thus the review by Calvo et al. [1] compiles the initial milestones of membrane research in Spain. It comments on the singularity of the first steps in membrane materials and mechanisms, by researchers from the field of Applied Physics and Irreversible Thermodynamics, mainly focused on ion separation processes. This was followed by applied disciplines such as chemical engineering, aimed at implementing membranes to improve existing or new industrial and environmental applications. These opened up myriad fields of knowledge in materials science, polymer chemistry, ceramic and inorganic materials, to design novel membrane materials to face the challenges experienced by foreign commercial membranes in practical applications. To highlight the increased attention accorded to membrane technology in Spain, this review also contains a directory of all the research groups active nowadays in membrane research in Spain.

In line with the origins of membrane technology studies in Spain and the current state of the art, among the Spanish articles included in this Special Issue, an important number of these articles report on the applications linked to the use of natural resources and the contribution of membranes to the solution of environmental problems related to this use.

Soto-Herranz et al. [2] investigated the use of gas permeable tubular membranes to optimise the reduction in ammonium from manure generated in livestock facilities. Using various e-PTFE membrane systems, NH_3_ in an acidic solution from a pig farm is extracted for its use as a fertiliser. In a second work, Soto et al. [3] continued this study by analysing the optimal conditions for NH_3_ capture in suspended gas membrane systems, analysing the usefulness of different capture solutions in terms of NH_3_ captured and economic cost.

In relation to the application of membrane processes to the partial dealcoholisation of wines, Calvo et al. [4] studied various alternatives for the partial dealcoholisation of white wines. They addressed the dealcoholisation of wines from Verdejo grapes by combining nanofiltration with pervaporation (PV), or single step PV, as well as a last possibility based on membrane dialysis. Of the three processes, dialysis performed best in terms of an adequate reduction in alcohol content, in the shortest time, and with the least disturbance to the organoleptic characteristics of the resulting wines.

The particularities of the production of lactic acid derivatives from the dairy industry were addressed by González Díaz et al. [5]. In their work, the pervaporation process was used for the dehydration of mixtures containing ethanol, ethyl lactate, and lactic acid, in addition to water, at high concentrations. Various membranes and the optimal conditions for the desired separation were analysed.

The growing interest in the production of biofuels has motivated numerous studies on separation techniques that allow the separation/concentration of organics produced by fermentation, improving productivity and performance. In this light, Arregoitia-Sarabia et al. [6] also studied novel pervaporation hollow fibre membranes composed of a PEBA layer and an external PDMS layer for the separation of butanol in order to evaluate their performance in the selective removal of organics from a synthetic ABE solution. In their study, they developed hollow fibres composed of a PEBA layer and an external PDMS layer, allowing an excellent selectivity to butanol (due to PEBA) together with the protective role of the PDMS layer, which allows sealing small holes or gaps in the fibres.

Regarding membrane modification, Jiménez-Robles et al. [7] modified the surface of PVDF membranes applied to the recovery of dissolved methane in aqueous streams. A three-step procedure (activation, functionalisation, and curing) allowed maximising the hydrophobicity of the starting PVDF membranes in order to increase the membrane performance in the dissolved methane recovery process.

The always interesting and active topic of membrane reactor research has also been addressed in this Special Issue through two articles from Spanish-based researchers. Particularly, Lejarazu-Larrañaga et al. [8], in an article selected as the Editor’s Choice by the journal’s editorial team, in a collaborative study performed in Portugal explored the application of reverse osmosis (RO) membranes in a process, patented by Portuguese researchers, known as Ion Exchange Membrane Bioreactor (IEMB). The specific application was nitrate removal from polluted water sources. For this purpose, end-of-life RO membranes were recycled, chemically modified and thus converted into anion-exchange membranes, which allowed for preparing them at potentially lower prices than those of commercial ones, also contributed to a more circular economy. Moreover, Yang and López-Grimau [9] addressed the development of a hybrid membrane bioreactor composed of a moving bed biofilm reactor combined with a membrane bioreactor (MBBR-MBR), applied to the treatment of wastewater from a Spanish textile company.

The presence in the Special Issue of several works dealing with the study of liquid membranes revealed the continuous interest in a promising and particularly active field of research in Spain. Firstly, León et al. [10] studied the kinetic and mechanistic modelling of emulsion liquid membranes. These models allowed to study the process of adsorption to the carrier-mediated transport in emulsion liquid membranes and their application to Cu(II) removal from aqueous solutions. On the other side, Alguacil and López [11] analysed the transport of iron (III) in mixed solutions obtained from the treatment of exhausted alkaline batteries by means of supported liquid membranes. In particular, the use of the carrier Cyanex 923, coupled to a liquid membrane with a flat solid support, allows the selective separation of Iron (III) from manganese (II) in solutions generated after the treatment of discharged batteries. Finally, Fontás et al. [12] used different ionic liquids as carriers for the surface characterisation of polymer inclusion membranes (PIMs). Techniques such as XPS or SEM were used to characterise the samples and estimate the possible losses of ionic liquids and the surface modifications performed. This last paper is certainly more related to membrane characterization, a field in which Spanish researchers have traditionally been very active, as can be seen in the Membrane Groups directory included in the Spanish-side review [1]. In this area, Lima-Rodríguez et al. [13] proposed a novel method for the non-destructive study of the mechanical properties of membranes. This procedure was based on the use of vibro-acoustic tests and the dynamic analysis of the behaviour of the membrane to these vibrational sound waves. This paper was also chosen as the Editor’s Choice.

Regarding the Portuguese-side contributions, the review by Tomé et al. [14] addressed the pioneering work on membrane science and technology in Portugal by the research groups of Instituto Superior Técnico—Universidade de Lisboa (IST), NOVA School of Science and Technology—Universidade Nova de Lisboa (FCT NOVA) and Faculdade de Engenharia—Universidade do Porto (FEUP) aiming to provide a historical perspective on the topic. Then, it overviewed the trends and challenges in membrane processes and materials involving Portuguese researchers, contributing to a more sustainable water–energy–material–food nexus.

The work by Janeca et al. [15] presented the synthesis and characterisation of novel cellulose acetate-based monophasic hybrid skinned amine-functionalized CA-SiO_2_-(CH_2_)_3_NH_2_ membranes using an innovative method that combines phase inversion and sol-gel techniques. The permeation properties of these membranes were assessed using an in-house-built single hemodialysis membrane module (SHDMM) under dynamic conditions. The CA-SiO_2_-(CH_2_)_3_NH_2_ membranes fully permeated urea, creatinine, and uric acid, while completely retaining albumin. Long-term filtration studies of albumin solutions indicated that fouling did not occur at the membrane surface.

Rita et al. [16] explored the use of membrane filtration to treat spent caustic effluents from Sines Refinery, in Portugal. These effluents are very challenging due to their very hazardous nature in terms of toxicity (rich in mercaptans, sulfides and other aromatic compounds) as well as their extreme pH (approximately 12–14). The potential of nanofiltration (NF) polymeric and ceramic membranes was assessed by membrane life expectancy. Although the polymeric membrane performance was very good, its lifespan was reduced after 6 weeks of contact with spent caustic effluents. Contrary to expectations, the ceramic membrane tested was not chemically more resistant than the polymeric one, suggesting that a pH of 13.9 is very aggressive, even for ceramic membranes.

We can summarize this Special Issue by concluding that, in the Iberian Peninsula, active membrane-related research is now being carried out in a variety of applications, ranging from the production of clean water for potable use, agriculture, and irrigation; to the recovery of valuable compounds from natural sources and industrial effluents; and membrane reactors to energy generation and storage, as well as the integration of membranes and membrane technology in carbon capture and utilization strategies. These cover a multi-scale approach from membrane materials synthesis to process development, involving fundamental science laboratories, chemical engineering departments and technological centres, and research institutes. As it has been shown in the articles composing the Issue, membrane research in the Iberian Peninsula is strongly concerned with Sustainable Development Goals and the evolution towards a sustainable and circular economy where membrane processes could be a unique tool.
